# Reports on the Hospitals of the United Kingdom

**Published:** 1912-03-23

**Authors:** Henry Burdett


### REPORTS ON THE

### Hospitals of the United Kingdom.

By SIR HENRY BURDETT, K.C.B., K.C.V.O.

### LXV.—Royal Infirmary, Sunderland.

This institution was founded in 1794, and it has been extended by the erection of additional wings. For, we believe, something like thirty years the nursing and domestic management of this infirmary have been practically in the hands of the Lady Superintendent, Miss Mary P. Thomson, who commenced her career at the Evangelical Protestant Deaconess' Hospital and Training Institution, Tottenham, the hospital portion of which institution has long since been merged into and absorbed by what is known as the Prince of Wales' General Hospital, Tottenham. For many years this institution had six deaconesses working at the Sunderland Infirmary, and there were still a few such sisters employed there when we inspected the hospital on September 19, 1911. Formerly Sister Mary discharged the duties of lady superintendent in an honorary capacity, but some years ago, the committee having decided to employ no unpaid labour for the future, she consented to the committee's wishes that she should be paid a salary.

### The Central Block needs Reconstruction. No Proper Staff Accommodation.

The Royal Infirmary covers a good deal of ground, and contains 216 beds, with an average occupancy of 184. Some of the pavilions in which the patients are housed are fairly complete, and the last and most pretentious of them exhibits some waste of money in unnecessary bricks and mortar, due to a want of knowledgeable thought in regard to details and measurements before the buildings were commenced. The oldest—that is, the central —block requires entire reconstruction from top to bottom. The present kitchens and offices, placed as they are on the ground floor, are inconvenient and out of date, and the sanitary blocks in connection with the oldest wards should be reconstructed and extended without delay. If this central block were modernised from top to bottom the expenditure need not be necessarily great, while the advantages to the hospital from an increase in its reputation, due to increased comfort for the patients and the provision of suitable quarters for the whole of the resident staff, including the medical officers, sisters and nurses, maids, and kitchen staff, would be considerable. It is a grievous thing to find, in a hospital which has been so frequently extended and has continued for so many years under the same direction, that there is at present absolutely no proper recognition of the claims of the resident staff of all grades to good hygienic quarters and accommodation. Why it is the Sunderland hospitals should stand out so conspicuously and unfavourably in regard to the accommodation, or rather the absence of accommodation, for the devoted workers who year after year live in the hospital atmosphere and devote their lives and energies to the cure of disease and attendance on the sick, it is impossible for a stranger to say. It may be that the accommodation for the employees of private residents in this town of Sunderland, or many of them, does not comprise adequate quarters for the servants. This may not, of course, be the case, but it seems to us to be intolerable that a great public hospital, under the supreme authority of a managing sister, should longer fail to recognise the paramount claims of the resident staff in this matter.

### Other Buildings Good but Incomplete.

Apart from the general criticism we have printed above, the plan on which the infirmary buildings have been constructed is far from convenient. It entails upon every member of the staff whose duty it is to be responsible for the hospital in its entirety, or to a considerable extent, the extra strain of going up and down stairs, in the absence of lifts, because every pavilion is kept distinct, and there is no gallery of communication between the first and other floors. The many inconveniences thus caused are apparently borne with patience and resignation by the staff, but they ought to be minimised or removed by the addition of several passenger-lifts, an urgently needed reform if the best interests of the patients and all those who serve them are to be the first consideration with the managers of this institution. The wards are good, especially in the newer portions of the hospital, but the waste in bricks and mortar is excessive, and in some pavilions the sanitary blocks, especially in the older wards, should be reconstructed and refitted. It appeared to us, both when we first arrived and subsequently, that the hospital has the air of considerable slackness in the general control, and greatly needs the appointment of a gate-porter of real ability to be placed in charge of the entrance and all that appertains to the smooth working of the many details devolving upon such an officer. The reconstructions here recommended are urgently called for, and we are glad to know that the cost of them is well within the means and resources of the institution. The balance-sheet shows that the total investments of all kinds exceed £116,000, of which £83,000 are invested in the land and buildings at present used by the infirmary.

### A New Children's Hospital.

The Royal Infirmary has erected at a cost of £21,000 a children's hospital with accommodation for fifty patients. This hospital stands on a new site, and consists of two pavilions of one storey each situated to the right and left of the administration block, which is placed in the centre, with the kitchens, etc., behind. The plan is a simple one, and this new departure should effect immense good to a large number of children resident within the area served by the Sunderland Royal Infirmary.

Sister Mary was unfortunately away when we inspected the Infirmary in September last. We are much indebted to the assistant matron, Miss Armour, who took infinite pains to explain and show us everything in detail. We noticed that the pharmacist, Miss Blyth, M.P.S., had her dispensary in excellent order and most conveniently arranged too.

